# mSphere of Influence: The bacterial growth law—revisiting the old to discover the new

**DOI:** 10.1128/msphere.00046-26

**Published:** 2026-06-23

**Authors:** Katsuya Fuchino

**Affiliations:** 1 Centre for Bacterial Cell Biology, Biosciences Institute, Newcastle University 5994, Newcastle upon Tyne, United Kingdom; Virginia-Maryland College of Veterinary Medicine, Blacksburg, Virginia, USA

**Keywords:** bacterial physiology, the growth law, *Zymomonas mobilis*

## Abstract

Katsuya Fuchino works in the field of bacterial cell biology and applied microbiology. In this mSphere of Influence article, he reflects on how the studies “Dependency on medium and temperature of cell size and chemical composition during balanced growth of *Salmonella typhimurium*” by Schaechter et al. and “A metabolic sensor governing cell size in bacteria” by Weart et al. inspired the field of bacterial cell size homeostasis and his current research. Additionally, he highlights the value of reading classic, original papers that influenced the field, as a means to reflect on paradigm shifts and generate new ideas.

## COMMENTARY

The nutrient growth law reported in 1958 stands as one of the most influential principles discovered in the field of bacterial physiology and possibly of bacterial cell biology (1, 2). The law beautifully captures bacterial growth and cell size regulation through simple and robust quantitative relationships: cell size increases exponentially as a function of the nutrient-imposed growth rate under steady-state conditions (1). The discovery laid the foundation for the field of bacterial cell size control, which has attracted interest from both experimental and theoretical microbiologists and physicists. Over the last 15 years, with the advent of advanced imaging techniques, powerful genetic tools, high-throughput approaches, and knowledge of bacterial cell organization at the molecular level, the field made significant progress (3–6). Now, we know that the original nutrient growth law may not strictly apply under certain conditions or at the single-cell level, and intrinsic stochastic effects are part of the size regulation (7). Currently prevailing models such as the “adder,” “timer,” and “sizer” models, along with their derivatives, have greatly advanced our understanding of size regulation. However, a fully integrated view of the field may not have been achieved.

The original study greatly inspired me and reminded me of the famous Confucian saying “revisiting the old to discover the new.” I was inspired by the study not only for its scientific and technical aspects but also for the philosophical aspects behind it, based on the following observations (and admittedly biased personal views). (i) The nutrient growth law might conceptually link molecular biology with system biology. As mentioned by the first author Moselio “Elio” Schaechter, the original study excluded reductionist approaches (8). However, the field apparently expanded through both systematic and molecular approaches. For example, the law was later revisited in light of new knowledge such as the tubulin homolog, key cell division protein FtsZ, and the replication initiator DnaA (3). (ii) Perhaps this was quite common at the time, but the used methodology was executed meticulously. Despite the relatively small sample size and the limited methods available at the time for measuring cell size, the authors were able to identify the correlation, which was indeed found to be robust at a population level using advanced imaging-based quantitative measurements (7). (iii) Apart from cell size, the growth law also revealed that cellular macromolecular composition is closely associated with nutrient-imposed growth rate. Although these two aspects of the growth law are interconnected (9), the topic of how bacterial cells allocate resources to their different macromolecules has its own biological implications, and thus, the law has far-reaching implications for bacterial physiology. (iv) The experiments were straightforward in leading to the discovery, almost as if the investigators had known the answer from the beginning. This study also originated from a classical observation that had already been known at the time. (v) Why did this study make such an impact? Interested readers are encouraged to read a personal note by the first author (8), which might offer hints toward future discoveries. In addition to scientific reasoning, I was particularly intrigued by his remark that the charm of Copenhagen may have influenced the work. This human aspect of working in science might become even more crucial in the era of artificial intelligence.

I was also inspired by another pioneering study that linked bacterial cell division/size and metabolism. The study by Weart et al. found that an enzyme, glucosyltransferase UgtP, responsible for the synthesis of glucolipids, also directly inhibits an assembly of a key division protein FtsZ in *B. subtilis*. As co-localization of UgtP with FtsZ is nutrient richness-dependent, the enzyme functions as a metabolic sensor to regulate cell size (10). The study inspired me because it is a combination of the topic of my doctoral and first postdoctoral training, but more crucially, I was impressed by how the authors identified the paradigm-changing regulatory mechanism through a relatively accessible approach. Thanks to several excellent studies, we now know that the cross-talk between metabolism (including both metabolic enzymes and metabolites) and cell morphology appears to be a conserved feature across several bacterial species (11–14), exemplifying how bacterial cells work as sophisticated and complicated living systems.

These two studies led me to speculate that there might be undiscovered functional links between cell size/morphogenesis and biological phenomena, which are currently considered unrelated. Whether this proves to be true or not, further understanding of the mechanisms underlying cell size regulation will likely be facilitated by technical advances. At the same time, I believe that the use of non-model organisms, especially the ones with distinctive biology, may provide an alternative approach. For example, the alpha-proteobacterium *Zymomonas mobilis* is acknowledged as one of the most efficient ethanol-producing microorganisms and possesses one of the simplest carbon metabolism and bioenergetics (15). The relatively simple physiology of *Z. mobilis* might provide a useful and unique experimental system to study how carbon metabolism and energy state influence cell size in fine detail. Interestingly, we recently found that engineering cell wall remodeling significantly influenced the size of *Z. mobilis* cells, reflecting the reduced functional redundancy within its genome. (16). Thus, the interplay between cell wall remodeling, metabolic alterations, and cell size regulation is worth investigating within this system.
